# VaProS: a database-integration approach for protein/genome information retrieval

**DOI:** 10.1007/s10969-016-9211-3

**Published:** 2016-12-23

**Authors:** Takashi Gojobori, Kazuho Ikeo, Yukie Katayama, Takeshi Kawabata, Akira R. Kinjo, Kengo Kinoshita, Yeondae Kwon, Ohsuke Migita, Hisashi Mizutani, Masafumi Muraoka, Koji Nagata, Satoshi Omori, Hideaki Sugawara, Daichi Yamada, Kei Yura

**Affiliations:** 1 Computational Bioscience Research Center, Biological and Environmental Sciences and Engineering, King Abdullah University of Science and Technology, Thuwal, 23955-6900 Saudi Arabia; 2 National Institute of Genetics, Shizuoka, 411-8540 Mishima, Japan; 3 Graduate School of Agricultural and Life Sciences, University of Tokyo, Bunkyo, Tokyo, 113-8657 Japan; 4 Institute for Protein Research, Osaka University, Suita, Osaka, 565-0871 Japan; 5 Graduate School of Information Sciences, Tohoku University, Miyagi, Sendai, 980-8597 Japan; 6 Tohoku Medical Megabank Organization, Tohoku University, Miyagi, Sendai, 980-8573 Japan; 7 Department of Maternal-Fetal Biology, National Research Institute for Child Health and Development, Setagaya, Tokyo, 157-8535 Japan; 8 Department of Pediatrics, St. Marianna University School of Medicine, Miyamae, Kawasaki, 216-8511 Japan; 9 Center for Informational Biology, Ochanomizu University, 2-1-1, Otsuka, Bunkyo, Tokyo, 112-8610 Japan

**Keywords:** Big data analysis, Database integration, Lysosomal storage disease, Protein 3D structure

## Abstract

Life science research now heavily relies on all sorts of databases for genome sequences, transcription, protein three-dimensional (3D) structures, protein–protein interactions, phenotypes and so forth. The knowledge accumulated by all the omics research is so vast that a computer-aided search of data is now a prerequisite for starting a new study. In addition, a combinatory search throughout these databases has a chance to extract new ideas and new hypotheses that can be examined by wet-lab experiments. By virtually integrating the related databases on the Internet, we have built a new web application that facilitates life science researchers for retrieving experts’ knowledge stored in the databases and for building a new hypothesis of the research target. This web application, named VaProS, puts stress on the interconnection between the functional information of genome sequences and protein 3D structures, such as structural effect of the gene mutation. In this manuscript, we present the notion of VaProS, the databases and tools that can be accessed without any knowledge of database locations and data formats, and the power of search exemplified in quest of the molecular mechanisms of lysosomal storage disease. VaProS can be freely accessed at http://p4d-info.nig.ac.jp/vapros/.

## Introduction

The advance of the molecular biology has yielded a huge amount of biological data including DNA/RNA/protein sequences [1–3], their expression levels [4], difference in the sequences of individuals [5], three-dimensional (3D) structures of the biomolecules [6], phenotypes of the organisms [7] and so forth. These data have been stored in independent databases located on the Internet and researchers exploit these databases for new knowledge of the target of their study. Database mining facilitates the process of knowledge acquisition and that of building new hypotheses for planning new experiments [8].

The expansion of the data size has been coped with the increase in the size of the storage and with invention of a new algorithm for searching the whole data swiftly. One of the famous examples of the tool for quick search of a database in this field is BLAST [9], a tool to search similar sequences out of the huge nucleotide/amino acid sequence databases. Further expansion of the size of the independent database and the increase in the variety of databases may have enhanced chances for performing novel experiments by extending the scope of hypotheses, yet the lack of technology for integrating different types of databases and of an application for searching the multiple databases have precluded extensive application of this approach. The researchers aiming for an integrated search of different databases should approach the databases one by one, learn how to use each database and obtain information relevant for their studies. The users then integrate the data obtained from different databases by themselves. This process evidently requires tedious labour as well as skills for manipulating data in different formats. Hence, the biggest hurdle that we have to overcome in the current life science activity is the complexity in integrating databases in a way that enables us to come up with novel ideas and hypotheses. Once the up-to-date data is comprehensively integrated, then researchers with an experience in a specific field can start deducing a hypothesis in a data-driven manner.

The effort for integrating the management of different databases has been made by a number of groups [10–14]. Linking data with a common framework is one of the possible approaches, and the Semantic Web technologies are becoming increasingly popular in recent years [15]. While the Semantic Web technologies based on linked open data and ontologies are a promising approach, extremely diverse set of ontologies as well as non-uniform uses of URI (Uniform Resource Identifiers) to describe identical resources by different parties make it difficult to integrate various information resources without extensive manual intervention. Although some efforts have been dedicated to solve these difficulties (e.g., http://identifiers.org), it will take time for the research community to agree on a unified convention.

To overcome these difficulties in a search of multiple databases in the information of life science, we started developing a new type of application that searches databases in different locations simultaneously by a simple search query and displays the result in a simple interface at http://p4d-info.nig.ac.jp/vapros/. We named the application VaProS, VAriation effect of PROtein Structure and function. The name derived from the aim of the application, namely to focus on analysing effects of DNA sequence variations on protein structures and function. VaProS aims to realize an idea of “data cloud”, that is to retrieve data without any knowledge of databases scattered in the Internet.

The idea embedded in VaProS that is different from other general database integration efforts is that VaProS makes much of the relationship among the biological molecules and phenomena. The relationship is governed by the central dogma; hence all the incidents can be described in either gene-centered or protein-centered manner. Phenotypic changes of an organism likely derive from changes in the biological system of the organism, which is sustained by the network of biomolecules and those biomolecules are ultimately encoded in DNA. This flow of information is nothing but the opposite direction of the central dogma, and hence the organization of data and databases in VaProS follows the information flow in the central dogma. Technically, the search results of the variety of databases are inter-connected using the protein as a hub of information. In the following sections, the detail of VaProS and the example of the usage are presented.

## Materials and methods

### Databases and tools on the internet

Table 1 lists the databases that are integrated on VaProS and the tools that visualize the search results. There are 16 databases and 15 tools of which VaProS are made. The latest information of the databases, namely the version and the size, is given at http://p4d-info.nig.ac.jp/vapros/statistics.html. The integration of the databases took the form of either dynamic link or a data copy from the original site to the local site of VaProS. Ideally, all the databases should be accessed dynamically to avoid time lag of the data and to save the local disk space, but such dynamic access often sacrifices a prompt response to a query. Therefore, we downloaded the part of the data from each database and achieved an optimum response speed. The data update is scheduled once in every six month to keep abreast with the latest data in all the databases. VaProS deals with the data of humans, rats and mice and focuses on phenomena related to humans.

**Table 1:** Components of VaProS

DB/tool name	Data resource	Search tool	Data/function used in VaProS	Method of access	Original location	Reference
EntrezGene	✓		Nomenclature, reference and other biological information of genes	Copy and link	http://www.ncbi.nlm.nih.gov/gene/	[16]
UniprotKB	✓		Amino acid sequences with biological annotation such as ontology and classification	Copy and link	http://www.uniprot.org/	[17]
BioGRID	✓		Genetic and protein interactions with curation based on biomedical literature	Copy and link	http://thebiogrid.org/	[18]
ChEMBL	✓		Drug-like small molecules with interacting proteins	Copy and link	https://www.ebi.ac.uk/chembl/	[19]
DrugBank	✓		Drug molecules combined with drug target informtaion	Copy and link	http://www.drugbank.ca/	[20]
IntAct	✓		Molecular interactions obtained from literature and direct submission	Copy and link	http://www.ebi.ac.uk/intact/	[21]
PID (NDEx)	✓		Biological interaction data of proteins	Copy and link	http://www.ndexbio.org/#/	[22]
Reactome	✓		Biological pathway data	Copy and link	http://www.reactome.org/	[23]
OMIM	✓		Mendelian disease related phenotype and its causative gene	Link	http://www.omim.org/	[7]
hGtoP	✓	✓	3D structural and comparative genomics annotations of humans, mice and rats	Link	http://p4d-info.nig.ac.jp/hGTOP/	[24]
Natural Ligand Database	✓	✓	3D models of proteins and their natural ligands registered in KEGG reaction database	Link	http://nldb.hgc.jp/nldb/	[25]
COXPRESdb	✓	✓	Relationship of gene expression based on RNAseq and microarray data	Link	http://coxpresdb.jp/	[26]
Mutation@A Glance	✓	✓	Genetic variants on proteins including disease-causing mutations observed in humans	Link	http://harrier.nagahama-i-bio.ac.jp/mutation/	[27]
3D Interaction	✓	✓	Models of protein 3D structure and the structure in complex with other molecules	Link	http://homcos.pdbj.org/	[28]
Autophagy DB	✓	✓	List of genes and proteins for autophagy	Built-in	http://www.tanpaku.org/autophagy/	[29]
GNP expression	✓	✓	Genes clustered by expression pattern showning co-regulation and anti-regulation	Built-in	http://genomenetwork.nig.ac.jp/	–
Molecular Interactions		✓	Graphic tool for interaction networks of proteins, compounds and phenotypes	Built-in	–	–
TagCloud		✓	Graphic tool to display frequency of words in the titles of papers registered in UniProt	Built-in	–	–
Pathway DB		✓	Finder of the related pathways from the databases in use	Built-in	–	–
Phenotype		✓	Finder of medelian disease related to the protein/gene in query	Built-in	–	–
Cis-finder		✓	Finder of the *cis* element candidate motifs in DNA sequence	Built-in	–	–
S-VAR		✓	Evaluator of the impact of missense mutation in a protein	Link	http://p4d-info.nig.ac.jp/s-var/	–
Genome explorer		✓	Annotator of genes with transcription start sites and other biological function	Built-in	http://genomenetwork.nig.ac.jp/	–
NOREN		✓	ID connector from UniProt AC to all the other IDs of the databases in use	Built-in	http://cib.cf.ocha.ac.jp/DC/	–

### Data integration and data presentation in VaProS

VaProS is unique in the style of data integration. VaProS tries to integrate different databases dynamically and relationship amongst the data in the databases is taken by UniProt accession key. Central dogma guarantees the relationship between biomolecules in the organisms, hence all the phenotypes should basically stem from the perturbation on biomolecules. Therefore, phenomena observed in the organization can be tagged to either DNA or protein. We chose a protein identifier to tag all the other data, because VaProS is aimed for the analysis of protein variation.

UniProtKB [2], GeneCards [12] and Cosmic [13] assume a similar approach for the integration of relevant data. VaProS put stress on a graphical presentation of the search results as found in “Molecular Interactions” and “TagCloud”, and on the analyses on protein 3D structures as found in “hGtoP”, “3D Interaction” and “Natural Ligand Database”.

### Search method

**Fig. 1:**
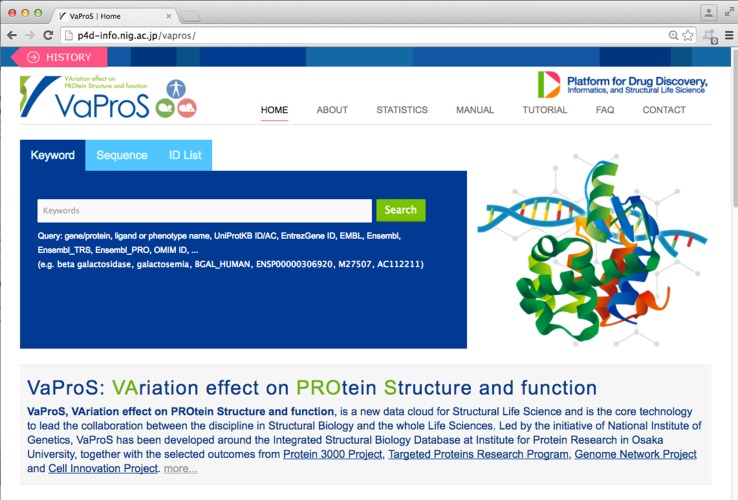
The top page of VaProS located at http://p4d-info.nig.ac.jp/vapros

**Fig. 2:**
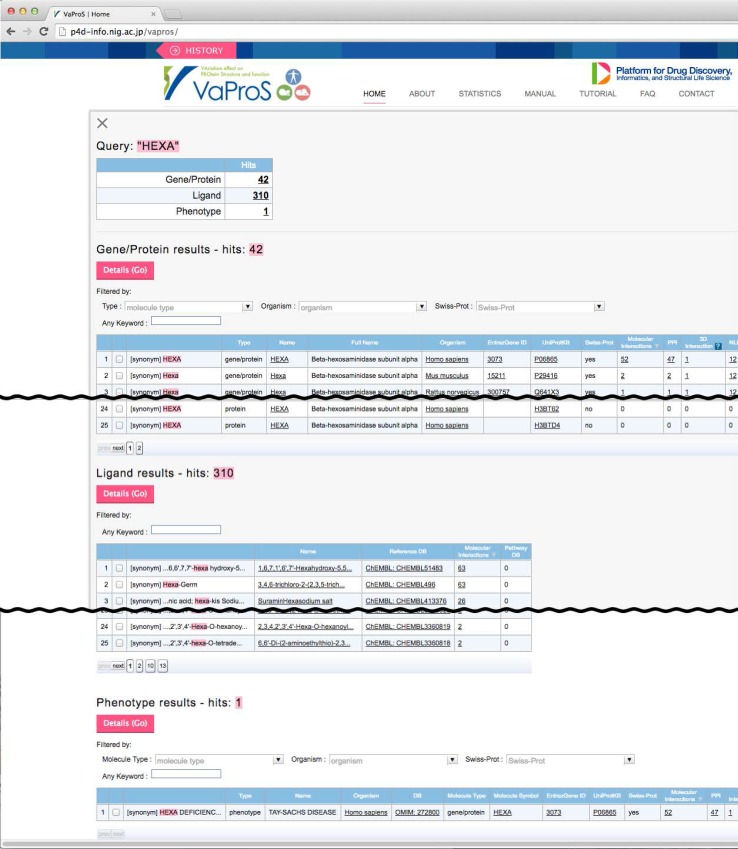
The initial search result by VaProS. The query word is “HEXA”, the causative gene of Tay-Sachs disease

**Fig. 3:**
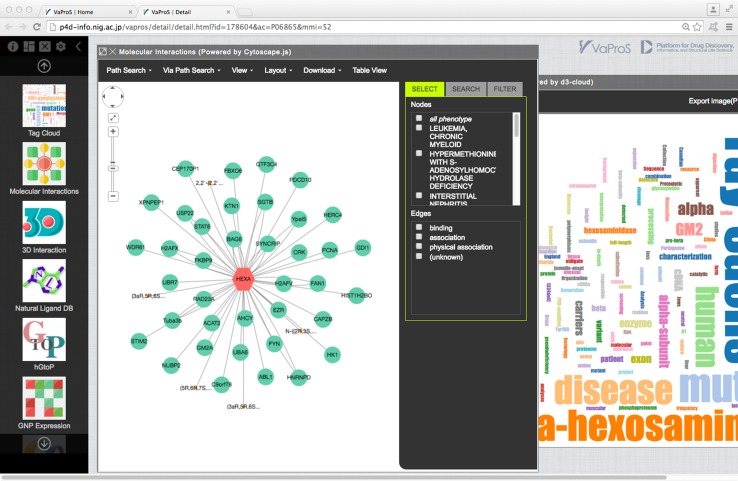
The search result in detail by pressing the “Details (Go)” button in Fig. 2. The protein–protein interactions and frequently used terms in literature related to HEXA are displayed

VaProS accepts keywords, DNA/protein sequence, EntrezGene ID and UniProtKB accession as a query (Fig. 1). A keyword can be a gene name, a protein name, a ligand (drug) name, a disease (phenotype) name and an identifier found in the databases. Input of the keywords is assisted by a keyword-suggestion function. Incomplete input makes VaProS find a related keywords in the keyword database and it shows a list of candidate words below the query input window. Once the Search button is pressed, VaProS throws the input data to NOREN, an original tool to search for whole IDs in the databases related to the query. NOREN is based on the ID mapping table provided by UniProtKB [2], and BLAST [9]. The result of the query is presented as a list of candidates to the user. The candidate list is categorized into three different types, namely Gene/Protein, Ligand and Phenotype (Fig. 2). The user may select the most relevant element in the list, press “Details (Go)” button and obtain the results of the search done by IDs relevant to the keywords (Fig. 3). The results are presented through the tools tabulated in Table 1. The search results shown by each tool can be opened by clicking the corresponding icon on the left in Fig. 3.

## Results and discussion

We explored the current knowledge on lysosomal storage diseases (LSDs) and built a tenable hypothesis as a case study to show the usage of VaProS. The similar analyses can be conducted on different diseases by accessing http://p4d-info.nig.ac.jp/vapros.

### Lysosomal storage disease

Lysosomes are subcellular organelles responsible for the physiological turnover of the cell constituents. They contain catabolic enzymes that require a low pH environment for their optimal function. LSDs are a heterogeneous group of more than 50 rare inherited disorders characterized by the accumulation of undigested or partially digested macromolecules (Table 2). LSDs ultimately result in cellular dysfunction and clinical abnormalities. LSDs are caused by deficiencies or defects in enzymes for lysosomes, in proteins necessary for the normal post-translational modification of lysosomal enzymes, in the activator proteins of lysosomal enzymes, and in the proteins important for proper intracellular trafficking between the lysosome and other intracellular compartments. The individual diseases are rare, but LSDs as a group affects many people around the world with a frequency of about one in every 7000–8000 live births [30, 31].

**Table 2:** Lysosomal storage diseases

Disease	Type	Gene	UniProt ID	PDB	PDB identity*
Mucopolysaccharidosis	IH (Hurler syndrome)	–	–	–	–
	IH-S (Hurler-Scheie syndrome)	IDUA	IDUA_HUMAN	3W81	100%
	IS (Hurler, Hurler/Scheie, Scheie syndrome)	–	–	–	–
	II (Hunter syndrome)	IDS	IDS_HUMAN	4UG4	36%
	III-A (Sanfilippo syndrome)	SGSH	SPHM_HUMAN	4MIV	100%
	III-B	NAGLU	ANAG_HUMAN	4XWH	100%
	III-C	HGSNAT	HGNAT_HUMAN	–	–
	III-D	GNS	GNS_HUMAN	4UG4	30%
	IV-A (Morquio syndrome)	GALNS	GALNS_HUMAN	4FDI	100%
	IV-B	GLB1	BGAL_HUMAN	3WF2	100%
	VI (Maroteaux-Lamy syndrome)	ARSB	ARSB_HUMAN	1FSU	100%
	VII (Sly syndrome)	GUSB	BGLR_HUMAN	1BHG	100%
	IX (Hyaluronidase deficiency)	HYAL1	HYAL1_HUMAN	2PE4	99%
Niemann-Pick disease	A	SMPD1	ASM_HUMAN	5FC5	35%
	B	–	–	–	–
	C1	NPC1	NPC1_HUMAN	3JD8	100%
	C2	NPC2	NPC2_HUMAN	2HKA	80%
GM1 gangliosidosis	I	GLB1	BGAL_HUMAN	3WF2	100%
	II	GLB1	BGAL_HUMAN	3WF2	100%
	III	GLB1	BGAL_HUMAN	3WF2	100%
GM2 gangliosidosis	Tay-Sachs disease	HEXA	HEXA_HUMAN	2GJX	99%
	Sandhoff’s disease	HEXB	HEXB_HUMAN	5BRO	98%
	AB variant	GM2A	SAP3_HUMAN	1PUB	100%
Sulfatide lipidosis	Metachromatic leukodystrophy	ARSA	ARSA_HUMAN	1N2L	100%
		ARSA	ARSA_HUMAN	1N2L	100%
	Multiple sulfatase Deficiency	ARSB	ARSB_HUMAN	1FSU	100%
		SUMF1	SUMF1_HUMAN	1Y1H	100%
Saposin dificiency	Prosaposin deficiency	–	–	4V2O	100% (fragments)
	Krabbe disease, atypical	–	–	3BQQ	
	Saposin B deficiency	PSAP	SAP_HUMAN	2DOB	
	Gaucher disease, atypical	–	–	1SN6	
Glycogenosis	II (Pompe disease)	GAA	LYAG_HUMAN	2QLY	47%
Gaucher disease	Gaucher disease	GBA	GLCM_HUMAN	2WKL	100%
Fabry disease	Fabry disease	GLA	AGAL_HUMAN	3LXB	99%
Ceramidosis	Farber’s disease	ASAH1	ASAH1_HUMAN	–	–
Krabbe disease	Krabbe disease	GALC	GALC_HUMAN	4UFH	84%
Cholesterol ester storage disease	Cholesterol ester storage disease	LIPA	LICH_HUMAN	1K8Q	60%
Wolman disease	Wolman disease				
Glycoprotein disorder	Alpha-fucosidosis	FUCA1	FUCO_HUMAN	2ZXA	39%
	Alpha-mannosidosis	MAN2B1	MA2B1_HUMAN	1O7D	83%
	Beta-mannosidosis	MANBA	MANBA_HUMAN	2VR4	31%
	Aspartylglycosaminuria	AGA	ASPG_HUMAN	1APZ	99%
	Galactosialidosis	CTSA	PPGB_HUMAN	1IVY	99%
	Mucolipidosis I	NEU1	NEUR1_HUMAN	1EUS	37%
	Mucolipidosis II	–	–	–	–
	Mucolipidosis III	GNPTAB	GNPTA_HUMAN	2N6D	99% (fragment)
	Schindler’s disease	NAGA	NAGAB_HUMAN	4DO4	99%
Membrane metabolism disorder	Cystinosis	CTNS	CTNS_HUMAN	–	–
	Sialic acid storage disease (Salla disease)	SLC17A5	S17A5_HUMAN	–	–
	Cathepsin K deficiency disease (pycnodysostosis)	CTSK	CATK_HUMAN	7PCK	100%
	Cobalamin F disease (cblF)	LMBRD1	LMBD1_HUMAN	–	–
	Danon disease	LAMP2	LAMP2_HUMAN	2MOM	100% (fragment)
Neuronal Ceroid Lipofuscinosis	Neuronal ceroid lipofuscinosis-1	PPT1	PPT1_HUMAN	3GRO	100%
	Neuronal ceroid lipofuscinosis-2	TPP1	TPP1_HUMAN	3EDY	100%
	Neuronal ceroid lipofuscinosis-3	CLN3	CLN3_HUMAN	–	–
	Neuronal ceroid lipofuscinosis-4A	CLN6	CLN6_HUMAN	–	–
	Neuronal ceroid lipofuscinosis-4B	DNAJC5	DNJC5_HUMAN	2CTW	100% (fragment)
	Neuronal ceroid lipofuscinosis-5	CLN5	CLN5_HUMAN	–	–
	Neuronal ceroid lipofuscinosis-6	CLN6	CLN6_HUMAN	–	–
	Neuronal ceroid lipofuscinosis-7	MFSD8	MFSD8_HUMAN	–	–
	Neuronal ceroid lipofuscinosis-8	CLN8	CLN8_HUMAN	–	–
	Neuronal ceroid lipofuscinosis-10	CTSD	CATD_HUMAN	2PSG	49%
	Neuronal ceroid lipofuscinosis-11	GRN	GRN_HUMAN	2JYE	100% (fragment)
	Neuronal ceroid lipofuscinosis-12	ATP13A2	AT132_HUMAN	3WGV	27%
	Neuronal ceroid lipofuscinosis-13	CTSF	CATF_HUMAN	1M6D	99%
	Neuronal ceroid lipofuscinosis-14	KCTD7	KCTD7_HUMAN	4UES	50% (fragment)
Congenital disorder of glycosylation	IA	PMM2	PMM2_HUMAN	2AMY	100%

*Amino acid sequence identity between the UniProt and PDB entries

### Search in the first step

**Fig. 4:**
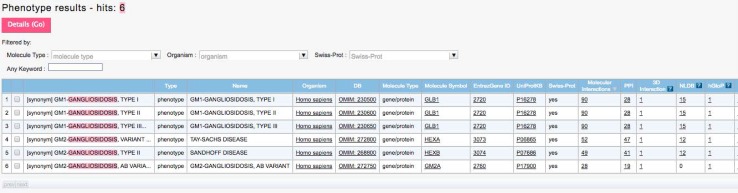
Initial search result by VaProS. The search by “gangliosidosis” initially results in a table of candidates

A search by a keyword “gangliosidosis”, one of the major groups in LSDs, resulted in six candidates as shown in Fig. 4. As shown in Table 2, gangliosidoses are classified into two types, GM1 and GM2, both of which are further classified into three subtypes. The estimated incidence of GM1-gangliosidosis is 1 per 100,000 to 200,000 births, and those of GM2-gangliosidosis are 1 per 360,000 births for Tay-Sachs disease and 1 per 310,000 or 1,000,000 births for Sandhoff disease. GM2-gangliosidosis AB variant is extremely rare [32]. Each line in the search result happened to correspond to an individual entry of genetic diseases/disorders in OMIM database [7]. The three types in GM1-gangliosidoses (types I, II, and III) were related to the same “Molecule Symbol”, namely GLB1 gene, but Tay-Sachs disease, Sandhoff disease and AB variant in GM2-gangliosidoses were related to HEXA, HEXB and GM2A genes, respectively. Each gene was linked to the databases listed in Table 1. Ticking the far left box in Fig. 4 and pressing “Details (Go)” button on the top led the user to the further detail of the selected item. In the following section, the search result of each tool listed in Table 1 is explained.

### Molecular interactions

**Fig. 5:**
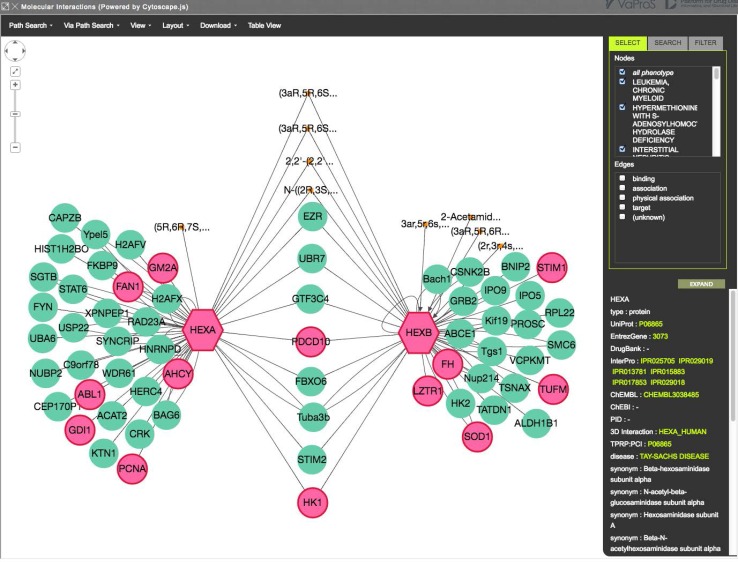
“Molecular Interactions” after selecting HEXA and HEXB in the initial search result (Fig. 4). A *big node* represents a protein, a *small node* represents a ligand and an *edge* represents a protein–protein/ligand interaction. A node in *red* is associated with a disease (selected in the *top-right* window)

The interaction network of the proteins encoded in HEXA and HEXB was found in “Molecular Interactions” window (Fig. 5). This window can be displayed by ticking both HEXA and HEXB in the table shown in Fig. 4 and pressing “Details (Go)” button. A protein is represented with a big node and a ligand is represented with a small node. A protein–protein/ligand interaction is represented with an edge. Figure 5 tells that eight proteins and four ligands interact both with HEXA and HEXB, and each protein interacts with a number of other proteins and ligands. These interactions were extracted from different databases listed in Table 1. In Fig. 5, the nodes in red are proteins associated with diseases. The information was extracted from OMIM (Table 1), and the catalog of specific disease is given on the right side of the window. There are two nodes in red that interact with both HEXA and HEXB, which suggest disease–disease interactions. By right clicking a node, protein–protein interactions can be extended. The pathway of two nodes in the window can be automatically detected using “Path Search” on the top menu.

By clicking a node or an edge, the detail information of the node/edge can be displayed on the right bottom of the window. In Fig. 5, HEXA was selected, hence the detail of HEXA was presented on the right. The link to “3D Interaction” shows the protein 3D structural information of HEXA protein. In 3D Interaction, SiteTable/SitesByVariants link leads the users to the information that VaProS aims for, namely the relationship between variations on DNA and protein structure/function.

### TagCloud

**Fig. 6:**
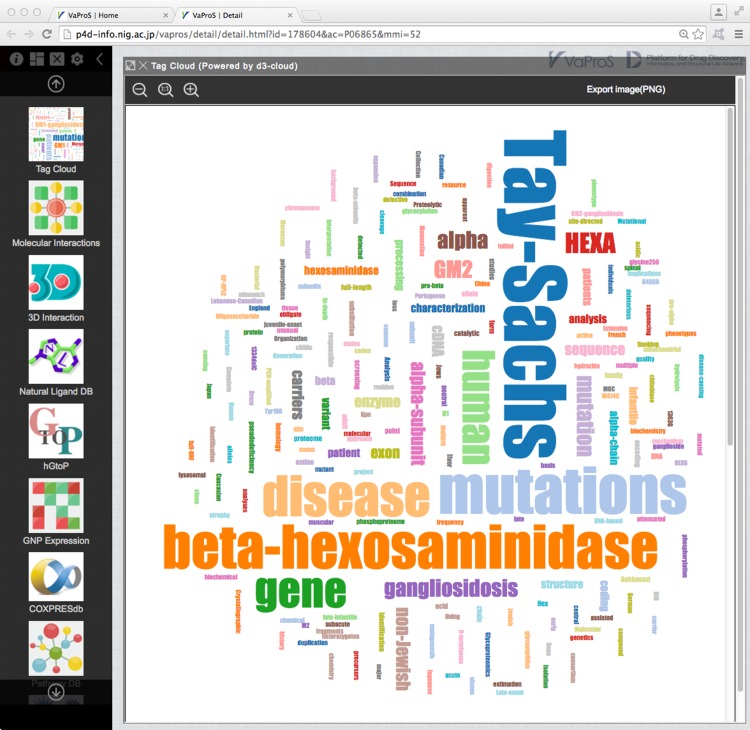
Artistic representation of the frequency of words in the titles of the manuscripts stored in the entry of HEXA_HUMAN in UniProtKB. The visualization was realized by d3-cloud (https://github.com/jasondavies/d3-cloud)

An overview of the target protein can be obtained by analyzing the frequency of words in the manuscripts related to the protein. Figure 6 is the result of such analysis on the titles of papers registered in UniProtKB under the entry HEXA. TagCloud emphasizes words that frequently appear in the titles of these papers by enlarging the size of the fonts. Visual inspection of TagCloud makes us recognize that HEXA protein is beta-hexosaminidase and may be a multimeric protein. TagCloud also ascertains that the protein is connected with the notion of disease. These facts are trivial for specialists in the field, but are not so for the researchers in different fields and are valuable information for the interdisciplinary study. The list of the papers using the word in the title can be found by clicking the word in the TagCloud.

### hGtoP

hGtoP provides relationship between genes and proteins as its name suggested (G in hGtoP stands for Gene/Genome and P for Protein). The original GtoP was developed by Kawabata et al. [24]. VaProS included human specific GtoP as one of the tools. With hGtoP, the structural information of the protein in the query is easily found. In addition, the homologues of the protein in different species can be found. “3D Interaction” and “hGtoP” contain similar information about protein 3D structures, however, the former focuses more on 3D modelling of complex structures, and the latter focuses on comparative genomics. PDB information in Table 2 was obtained by hGtoP and 3D Interaction. The information clarified that some of the proteins in LSDs do not have structural information yet. In other words, Table 2 provides valuable information for structural genomes of LSDs, namely the target proteins for determining 3D structures.

### Natural ligand database

Natural Ligand Database (NLDB) [25] provides the model of protein structures with natural ligands. The idea stemmed from the fact that many ligands in PDB are modified ligands for the sake of crystallisation and the bridge between those modified ligands and natural ligands should be provided to enhance the 3D structural information in PDB. The search query “GM1 gangliosidosis” led the user to the causative gene GLB1. NLDB demonstrated that GLB1 was involved in 15 KEGG reactions, and these 15 reactions were classified into five pathways according to “UniProt search view”. The same 15 reactions can be found in “Pathway DB” tool (Table 1). Of the five pathways in NLDB, glycosphingolipid biosynthesis (hsa00604) contained the reaction of GM1 degradation (R06010). In this reaction, 56 natural ligand complexes were registered in NLDB derived from the proteins of various species. The link to human beta-galactosidase, the product of GLB1 gene, with galactose (PDB ID: 3THC) led to the 3D structures of the ligand–protein complex with reported variation in amino acid residues. The variations in amino acid residues around the ligand-binding site were highlighted on the table of NLDB window. In this entry, ten variations were reported around the ligand-binding site, and eight of them were related to diseases, namely three to GM1 type I, one to GM1 type II, one to GM1 type III, and two to mucopolysaccharidosis IV-B.

### COXPRESdb

Gene co-expression sometimes sheds light on relationship between genes, and COXPRESdb [26] provides user-friendly interface to gene co-expression information in humans, mice and rats. The search query “GM2 gangliosidosis” on VaProS led the user to a list of causative genes that included HEXA and HEXB. COXPRESdb demonstrated that the co-expression for HEXA (PCC = 0.43) and HEXB (PCC = 0.45), which are known to be related with Sandhoff disease and Tay-Sachs disease, respectively from OMIM information. By following the link to COXPRESdb, the user can also check the co-expression networks of HEXA and HEXB, which led to the finding that ten more lysosomal proteins are tightly co-expressed with them.

### Other tools

GNP Expression, Phenotype, Autophagy DB, Genome Explorer and Mutation@A Glance (Table 1) show the search results in a tabulated or graphical form. The user can further analyze the database of each tool by following the link in the search result.

S-VAR is a special tool that evaluates impact of amino acid substitution to the function of the protein. By providing a specific mutation to the window of S-VAR, the tool starts a couple of software that evaluate the impact of the mutation [33–36] and provides each and consensus results for the user at some intervals.

### Building hypothesis on the relationship between phenotype and protein 3D structure

Combination of the search results in each tool can be a basis of building a hypothesis that can be verified by wet-lab experiments. The search query “GM1 gangliosidosis” on VaProS led the user to the current knowledge that the causative gene is GLB1, which encodes lysosomal enzyme β-d-galactosidase. By following the link to OMIM [7], user can acquire information on the detail of the disease, namely GM1 gangliosidosis is classified into three types in accordance with the onset age and severity; type I (infantile), type II (juvenile) and type III (adult) as shown in Table 2.

**Fig. 7:**
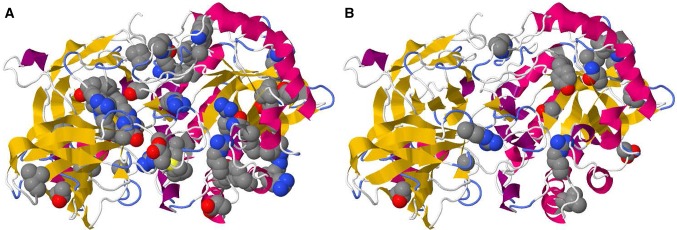
Variations of GM1-gangliosidosis mapped onto the protein 3D structure of GLB1. Variations in type I (**a**) and variations in type III (**b**). The structure of human β-galactosidase (PDB ID: 3WF2) is used for the mapping

“3D Interaction” summarized the variations leading to each type of diseases on the protein 3D structure (Fig. 7). Visual inspection of the figure tells that the variation tends to be located inside the protein. Indeed, the ratios of buried residues for type I was 95.5%, for type II was 88.2%, and for type III was 86.7%. These ratios are significantly higher than the average of the protein (Table 3). Note that the ratio of buriedness of the variation site is highest in type I and lowest in type III. Generally, mutations on the buried sites often make the protein less stable than the native one. Hence the observation suggests that the variations on type I have more impact on the stability of the protein than those on type III. 3D Interaction also provided amino acid frequencies of homologous proteins. The mutation to the rare amino acid implies that the type of amino acid has not sufficiently fixed during the molecular evolution. The ratios of mutations to the amino acids that no homologues used were 78.4% (type I), 73.7% (type II) and 53.3% (type III). Serious phenotypes are expected by mutations to amino acid types rarely observed in homologues. The buriedness and trends in amino acid types between types I and III apparently correlated with the degree of seriousness in each type of GM1 gangliosidoses. The similar trend was discussed by Ohto et al. at the time they determined the 3D structure of the protein [37]. VaProS enables such complex hypothesis building in a short period of time.

**Table 3:** Summary of the mutated sites of GLB1 on protein 3D structure for GM1-gangliosidosis

	GM1 type I	GM1 type II	GM1 type III	All residues
Number of residues	45	17	15	677
Buried residue^a^ (%)	95.5	88.2	86.7	56.8
Exposed residue^b^ (%)	4.5	11.8	13.3	43.2

^a^Residue with relative solvent accessibility less than 20%.

^b^Residue with relative solvent accessibility no less than 20%.

## Conclusion

Here we launched VaProS, a new type of database integration application. VaProS enables a quick search of multiple databases with interrelation of each search result. This application can be used as a textbook for acquiring expert knowledge for researchers in different fields, and can be a tool for building a data-driven hypothesis that can be tested by wet-lab experiments [16–23].
